# Learning selection-based augmented reality interactions across different training modalities: uncovering sex-specific neural strategies

**DOI:** 10.3389/fnrgo.2025.1539552

**Published:** 2025-04-28

**Authors:** John Hayes, Joseph L. Gabbard, Ranjana K. Mehta

**Affiliations:** ^1^Department of Industrial and Systems Engineering, Texas A&M University, College Station, TX, United States; ^2^Grado Department of Industrial and Systems Engineering, Virginia Tech, Blacksburg, VA, United States; ^3^Department of Industrial and Systems Engineering, University of Wisconsin Madison, Madison, WI, United States

**Keywords:** augmented reality, fNIRS, psychomotor learning, sex differences, graph theory, training, observational motor learning, motor practice

## Abstract

**Introduction:**

Recent advancements in augmented reality (AR) technology have opened up potential applications across various industries. In this study, we assess the effectiveness of psychomotor learning in AR compared to video-based training methods.

**Methods:**

Thirty-three participants (17 males) trained on four selection-based AR interactions by either watching a video or engaging in hands-on practice. Both groups were evaluated by executing these learned interactions in AR.

**Results:**

The AR group reported a higher subjective workload during training but showed significantly faster completion times during evaluation. We analyzed brain activation and functional connectivity using functional near-infrared spectroscopy during the evaluation phase. Our findings indicate that participants who trained in AR displayed more efficient brain networks, suggesting improved neural efficiency.

**Discussion:**

Differences in sex-related activation and connectivity hint at varying neural strategies used during motor learning in AR. Future studies should investigate how demographic factors might influence performance and user experience in AR-based training programs.

## 1 Introduction

Recent advances in immersive technologies have made augmented reality (AR) more readily available and affordable (Yin et al., 2021), with continued growth expected in the coming years. Virtual reality (VR) has already made its way into many industries, with commercially available VR-based training applications already on the market (Bric et al., 2016; Hayes et al., 2022), but AR offers some distinct advantages over VR. Unlike virtual reality, which completely immerses the user in a virtual environment, AR places virtual objects within the real world and allows the user to interact with both real and virtual objects. This opens the possibility for AR to be used for realistic training simulations in naturalistic environments rather than fully simulated virtual environments. Additionally, unlike VR, AR is not limited to training but could also be deployed for use in the field (Braly et al., 2019). AR has been shown to have potential applications in a wide range of industries, including healthcare (Gerup et al., 2020), construction (Rankohi and Waugh, 2013), emergency response (Sebillo et al., 2016), and manufacturing (Nee et al., 2012).

However, despite the potential benefits that AR offers, it is a novel interface requiring a new set of skills and interactions distinct from current widespread technologies, such as touchscreens. Prior studies have indicated that selection-based interactions in AR involve a learning curve, which can lead to some difficulty and frustration before they can be effectively utilized to enhance user performance (Dwivedi et al., 2022). Therefore, some form of AR training or familiarization will be necessary before it can be successfully implemented and enhance performance in training or on the field (Vyas et al., 2024). This raises unresolved questions about the most effective and efficient methods for delivering this training. For instance, would a brief video illustrating basic selection-based AR interactions suffice, or is hands-on practice with the AR headset more effective for familiarizing users with the interface? At the heart of this issue lies a more fundamental knowledge gap regarding the distinctions between observational motor learning and hands-on AR-based motor learning.

Neuroimaging studies have shown that both motor observation and motor execution enhance motor learning through a shared neural network, specifically the Action Observation Network (Jeannerod, 2001; Balconi et al., 2017). The AON involves the premotor cortex, supplementary motor area, and the primary motor cortex and is associated with motor learning and automaticity (Toni et al., 1998; Debaere et al., 2004; Wu et al., 2004). Prior studies have shown that motor learning through physical execution elicits higher activation of these brain regions when compared to observational learning (An et al., 2013; Su et al., 2023). Additionally, prior studies have reported that males benefit more in gaining procedural motor skills than females from motor training, and differences in neural structures and strategies may explain these sex differences (Kennedy and Raz, 2005; Dorfberger et al., 2009). These studies support our hypothesis that enhanced neural strategies may be more prominent with hands-on AR-based motor learning than with observational motor learning through video-based demonstrations, and that these strategies may differ by sex. It is important to note that these neuroimaging studies focused on basic hand-grasping actions, in contrast to the selection-based motor interactions used in AR. Therefore, the effect of cognitive load associated with different interaction modes (e.g., poking, raycasting, scrolling, moving) should also be considered when evaluating various training modalities for AR interfaces.

AR offers the benefit of being more immersive than other traditional systems, such as video instruction, and it is known that immersion can improve learning (Dede, 2009) and reduce cognitive load (Psotka, 1995). AR has been previously shown to improve declarative learning and visualization (Chen, 2008; Radu, 2012), and AR-based instructions were shown to improve the performance of a real-world (i.e., non-virtual) procedural task compared to traditional instructions (Henderson and Feiner, 2011; Braly et al., 2019). It is thus expected that learning a selection-based task by practicing it virtually in AR would be more effective than learning by watching a video. However, the effectiveness of AR as a motor learning tool (i.e., specifically practicing a virtual task in AR) is inconsistent in the literature, and AR has not always been found to be more effective than other methods (Dwivedi et al., 2022). It has been shown that the design of an AR interface can significantly impact the mental and physical demands and performance of a user during an AR-based motor activity (Kia et al., 2021). Further, the use of AR and other head-mounted displays (HMD) also presents possibilities of increased cognitive demand, nausea, motion sickness, and other adverse effects (Gavgani et al., 2017; Kia et al., 2021). Finally, higher immersion is not always necessary for effective learning (Bowman and McMahan, 2007). For example, it is unclear if all or some interactions (e.g., poking vs. raycasting) will benefit from AR-based training or if some training could be effectively conducted with more cost-effective video-based training. Therefore, there is a need to understand the fundamental nature of motor learning within AR and how it differs from other traditional training tools to employ it effectively.

The current body of literature regarding augmented reality (AR) has primarily concentrated on user experience and performance; however, there exists a paucity of research addressing potential sex differences that may influence these outcomes (Bend and Öörni, 2023). A limited number of studies have investigated the presence of sex differences in AR interactions, indicating that males typically excel over females in competencies such as spatial visualization, orientation tasks, and navigation (Waller et al., 1998; Ahmad et al., 2005). Furthermore, Yan et al. (2021) reported that women experienced a heightened cognitive load compared to men during an AR-based warehousing task, yet demonstrated more rapid enhancements in operational efficiency relative to their male peers. Considering that motor execution and learning are susceptible to the influence of sex differences (Cohen and D'Esposito, 2016), a comprehensive analysis of sex differences in selection-based AR interactions is warranted.

The goal of this study was to explore the fundamental differences between observational video-based motor learning and hands-on AR-based motor learning by examining how these modalities differentially affect a user's performance and perceptions of workload, cognitive load, and engagement. A secondary goal was to evaluate any potential sex differences in these outcomes. To compare the effectiveness of the two training modalities, we analyzed user performance during an evaluation task within the AR environment that followed either AR or video-based training. To assess user experience and the cognitive load imposed by each form of training, we compared subjective workload ratings from both the training and evaluation phases. Finally, we employed a neuroergonomics approach to investigate how each training modality impacts the neural strategies users utilize following training, as this can provide fundamental insights into differences in performance and skill acquisition. Examining how the brain recruits various regions associated with distinct functions (e.g., working memory, attention, or motor preparation and execution) during an AR-based motor learning activity could offer critical insights into AR-specific cognitive demands placed on users.

## 2 Methods

### 2.1 Participants and experimental protocols

Thirty-three participants (17m/16f) were recruited from a university community. All participants reported being right-handed and attested to having <1 h of combined AR and VR experience to minimize any confounds related to prior AR experiences. The participants ranged in age from 19 to 59 years (mean: 23.2; SD: 7.2 years). Each participant provided informed consent under the approval of the Texas A&M Institutional Review Board (IRB2021-0742).

Participants were randomly assigned into one of two groups: AR (8m/8f) and video (9m/8f). The experiment consisted of a training phase and an evaluation phase. Upon arrival at the lab, participants were equipped with a functional near-infrared spectroscopy (fNIRS) device (NIRSport2, NIRx Medical Technologies LLC, NY, USA.), and a three-minute resting baseline (seated with closed eyes) was collected in a quiet, dark room. Participants then completed training on four basic AR interactions: poking, raycasting, scrolling, and moving. These interactions were chosen because of their wide utility in a broad range of AR applications. Poking and moving are generally regarded as simpler interactions, while raycasting and scrolling are more challenging, requiring more control and coordination. Poking is used to select nearby objects that are within arm's reach of the user, by touching the object with a finger. Raycasting is used to select objects that are outside of the arm's reach by directing a ray that extends from the palm of the user's hand toward the object and tapping the thumb and forefinger together. Scrolling uses raycasting to scroll through a list of items on a virtual menu and select the correct item. Moving uses raycasting to select and hold onto a virtual object and move it to a different location.

During the training phase, participants in the AR group practiced each interaction with the Microsoft HoloLens 2 AR headset (Microsoft Corp., Redmond, WA). The order of the interactions was counterbalanced between poking and raycasting and between moving and scrolling, with poking/raycasting always being presented before moving/scrolling. At the beginning of training for each interaction, written and verbal instructions on how to perform the interactions were provided to the participant through the AR headset. The poking interaction required the user to use the poking interaction to select virtual buttons labeled from one to ten. The buttons were arranged in two rows and five columns within arm's reach of the participant, but the order of the numbers was randomized. The raycasting interaction required the participant to use the raycasting interaction to select virtual buttons labeled from one to ten. The buttons were in the same arrangement as the poking interaction, but they were located outside of the arm's reach. The scrolling interaction required the participant to use raycasting to scroll through a virtual menu with a list of names of types of fruit and select a specific fruit. The moving interaction required the participant to use raycasting to move a small virtual box inside a larger virtual box. Participants completed six consecutive trials of each of these interactions, with a two-minute rest period in between the different interactions. For all interactions, the participant was instructed to use only the right hand and was asked to complete each interaction as quickly and accurately as possible.

Participants in the video group were trained on the four interactions by watching four videos on a computer monitor demonstrating each of them. Each video lasted 25–45 seconds and demonstrated one of the four interactions. The videos began with written and verbal explanations of the interactions, followed by a demonstration of the interaction from the viewpoint of the user within the AR headset. The counterbalancing and rest breaks were similar to those employed in the AR group.

After training, both groups completed the evaluation phase, which involved performing three consecutive trials of each interaction within the AR headset. Participants were encouraged to complete each trial as quickly and accurately as possible, with a two-minute rest period in between the different interactions.

### 2.2 Measurements

#### 2.2.1 Performance

The time of completion for each interaction during evaluation was used as a measure of performance. Completion times of each trial were averaged across trials for each interaction. We chose time to complete as the performance metric over accuracy as the intent of the training was to enhance motor skill acquisition with the four AR interactions (Vyas et al., 2024).

#### 2.2.2 Subjective responses

Participants completed the NASA Task Load Index (NASA-TLX) (Hart and Staveland, 1988; Hart, 2006) and the Cognitive Load Theory (CLT) (Klepsch et al., 2017) surveys after each interaction in the training and evaluation phases. For the NASA-TLX survey, participants were asked to respond to six questions on mental demand, physical demand, temporal demand, effort, frustration, and performance on a scale from one (Low) to twenty-one (High). The unweighted total NASA-TLX score for each interaction was calculated by taking the sum of all the sub-scores (Moroney et al., 1992). A previous study used NASA-TLX to measure subjective workload during a motor learning task of varying difficulty levels and found that maximal improvement occurred at a moderate workload level and decreased under very high and very low workloads (Akizuki and Ohashi, 2015). Therefore, comparing subjective workload during training in AR and video modalities could help to explain any performance differences during evaluation. Further, identifying the workload associated with each modality and each interaction can help to calibrate the workload level of future training to elicit maximal learning. For the cognitive load theory survey, participants were asked to rate on a scale from one (Low) to seven (High) how much they agreed with eight statements on the germane, intrinsic, and extraneous cognitive load of the interaction (Klepsch et al., 2017). Cognitive load theory provides more detailed insight into the specific cognitive demands associated with a task, dividing cognitive load into three categories: intrinsic, extraneous, and germane. Intrinsic load depends on task complexity. Extraneous load depends on the design of the learning task or learning environment. Germane load is related to the engagement of mental processes that facilitate learning and the development of mental models (Klepsch et al., 2017). This can help to distinguish beneficial load from non-beneficial cognitive load to optimize the efficiency of a training task (Paas and van Merriënboer, 2020). Germane load, intrinsic load, and extraneous load sub-scores were calculated from CLT scores, as described previously (Klepsch et al., 2017).

Participants also completed the User Engagement Survey (UES) (O'Brien et al., 2018) at the end of the training phase and the end of the evaluation phase, in which they were asked to rate from one (Low) to five (High) how much they agreed with 12 statements on the usability, aesthetic appeal, and engagement of the task. Engagement is an important factor to consider when designing a learning interface to improve the experience of the user and facilitate learning (O'Brien and Toms, 2008). Engagement has previously been found to correlate with performance (Askari et al., 2020), and the UES has been used previously to compare engagement across different learning platforms (Lopez-Ozieblo et al., 2021). Identifying differences in user engagement during AR vs video training could help to explain performance differences. It could also help to provide a better understanding of the user experience associated with this new form of training, which could be important in the implementation of this tool in the future. Four metrics—aesthetic appeal, focused attention, perceived usability, and reward—were calculated from the UES scores (O'Brien et al., 2018).

#### 2.2.3 fNIRS measures

**Figure 1:**
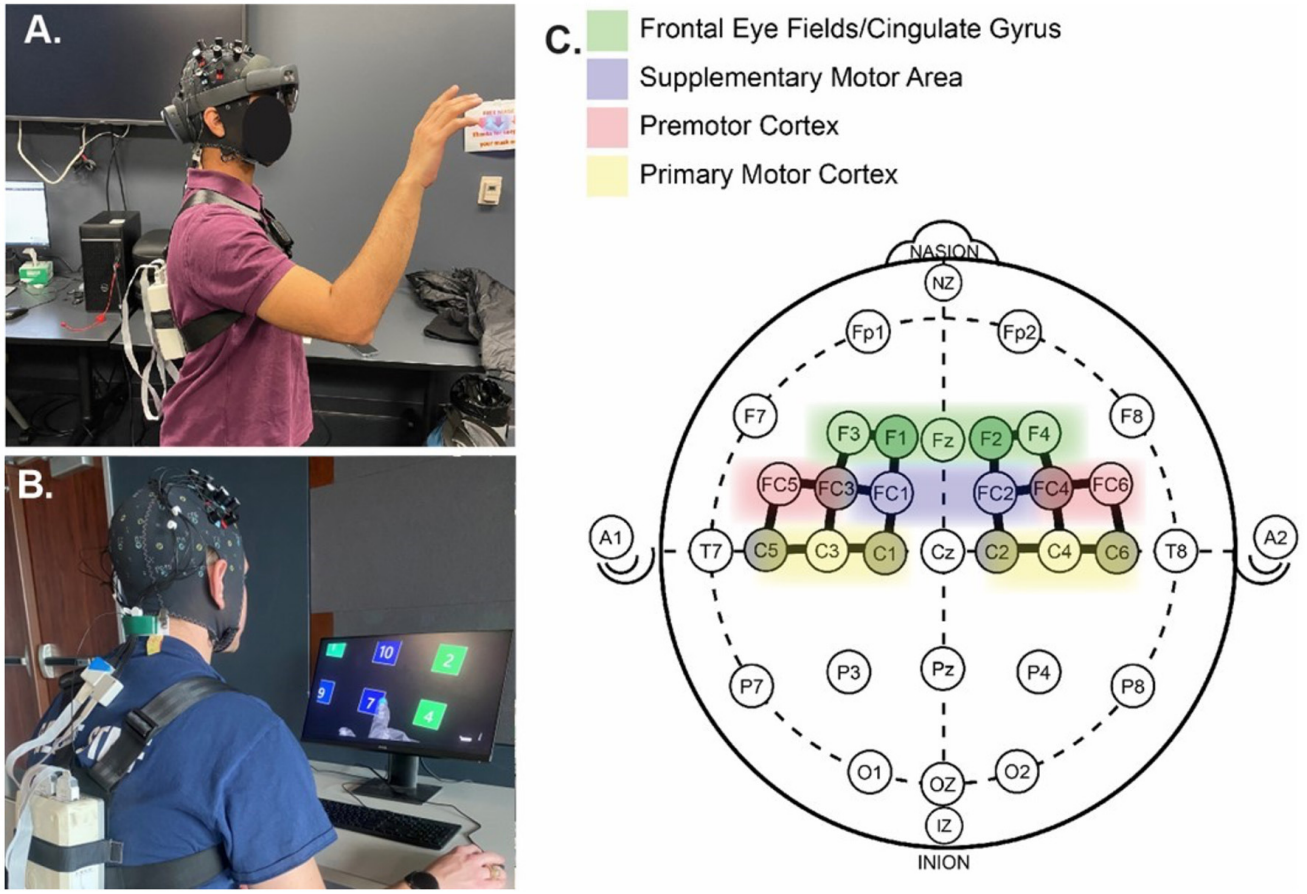
**(A)** AR training, **(B)** video training, **(C)** fNIRS probe montage: sensors (white) and detectors (gray) are connected by solid black lines indicating the channels.

An fNIRS system was used to measure the hemodynamic response of the brain during the training and evaluation tasks. fNIRS uses the concentration of oxygenated hemoglobin in the blood as a functional indicator of brain activity (Zhu et al., 2019). An 8 × 8 fNIRS system (NIRSport2, NIRx Medical Technologies LLC, NY, USA.) was employed using a probe map on the International 10–20 Coordinate System (Jasper, 1958) consisting of 20 channels and targeting six brain regions: Frontal Eye Fields/Cingulate Gyrus (FEF/CG), Supplementary Motor Area (SMA), Left Premotor Cortex (LPMC), Right Premotor Cortex (RPMC), Left Primary Motor Cortex (LM1), Right Primary Motor Cortex (RM1) (Figure 1). These regions were monitored based their involvement in motor function and on prior literature which identified changes in the activation of these regions associated with the AON for motor learning and automaticity (Toni et al., 1998; Debaere et al., 2004; Wu et al., 2004). The prefrontal cortex has also been shown to play a role in motor learning (Wu et al., 2004; Alves Heinze et al., 2019); however, the position of the AR HMD interfered with the placement of probes in the prefrontal regions, so prefrontal cortex activity could not be collected during this study. At the start of the experiment, the fNIRS cap was positioned on the participant's head using the Cz and Fpz, based on the International 10–20 Coordinate System (Jasper, 1958), as landmarks to ensure accurate probe placement. The participant was fitted with an opaque, black shower cap to block outside light from interfering with the measurement, and a signal optimization test was performed in the NIRx Aurora software (Aurora fNIRS, NIRx Medical Technologies LLC, NY, USA). After optimization, a three-minute baseline measurement was recorded, during which time the participant was asked to remain seated and still with eyes closed.

fNIRS data was processed using the MATLAB toolbox Homer2 (Huppert et al., 2009). First, the raw intensity data was converted to optical density. Next, motion artifact correction was applied by a kurtosis-based wavelet algorithm with a kurtosis threshold of 3.30 (Chiarelli et al., 2015). Then, a hybrid spline-SG motion correction was applied (Jahani et al., 2018). A bandpass filter was applied to remove artifacts from respiration and heartbeat as well as low-frequency waves (Alves Heinze et al., 2019) with a high pass filter frequency of 0.010 Hz and a low pass filter frequency of 0.50 Hz (Shi et al., 2020; Tyagi et al., 2021). Finally, a modified Beer-Lambert law was used to convert the optical density into concentrations of oxygenated (HbO) and deoxygenated (HbR) hemoglobin (Delpy et al., 1988; Rhee and Mehta, 2018). HbO is a better indicator of cerebral blood flow than HbR, so it was used for the remaining analysis (Hoshi et al., 2001). Brain activation and functional connectivity metrics were extracted from the task-related HbO signals during the evaluation phase.

Brain activation has been measured in prior motor learning studies using both fMRI and fNIRS, and distinct changes in activity have been identified in association with motor learning (Toni et al., 1998; Debaere et al., 2004; Wu et al., 2004; Alves Heinze et al., 2019). These changes are associated with the development of automaticity (Wu et al., 2004), and therefore, comparing brain activation patterns in the two groups during evaluation can help to capture differences in the effectiveness of motor learning within each modality.

Activation was calculated for each interaction during evaluation as described in Shi et al. (2022). The start and end times of each trial were extracted from the AR headset log and were used to extract the HbO concentrations corresponding to that period. For every channel, mean activation was calculated for each trial by taking an average of the HbO concentration during that trial (Shi et al., 2022). A global baseline was calculated by taking the average HbO concentration during the second two minutes of the three-minute baseline period, and the mean activation for each trial was corrected by subtracting this baseline. Channels were grouped into the six regions of interest (namely, FEF/CG, SMA, RPMC, LPMC, RM1, and LM1; displayed in Figure 1), and region of interest (ROI) averages were calculated for each trial. The mean value of all the trials for each interaction was taken as the mean brain activation for that interaction.

Functional connectivity can provide additional insight into the neural processes associated with a task. Functional connectivity examines the correlations between the HbO time series of each channel to identify brain networks of coordinated activity (Rogers et al., 2007; Tyagi et al., 2021). Quantifying the number of connections within and between different brain regions can provide information about the network recruited for the task and the degree of communication between different brain regions.

Using the start and end times from the AR headset, the HbO concentration curves for the four interactions during the evaluation were spliced together into a single continuous curve for each channel (i.e., the rest periods between interactions were removed). The Pearson Correlation coefficient between each pair of channels was calculated for each participant. Channel pairs with a Pearson Correlation coefficient between −0.291 and 0.291 were considered spurious and were removed. This threshold, determined via Monte Carlo simulation, sets the 99th percentile for *p* < 0.01 to reduce false positives, ensuring only the strongest correlations are seen as significant (Hocke et al., 2016). For each participant, the number of non-spurious connections within the motor regions (LM1, RM1), within the frontal regions (FEF/CG, SMA, RPMC, LPMC), and between the motor and frontal regions was determined. Owing to the short duration of each interaction, functional connectivity analysis was pooled across all four interactions based on the recommended duration (~3–5 minutes) of analysis (Zhu et al., 2019).

**Figure 2:**
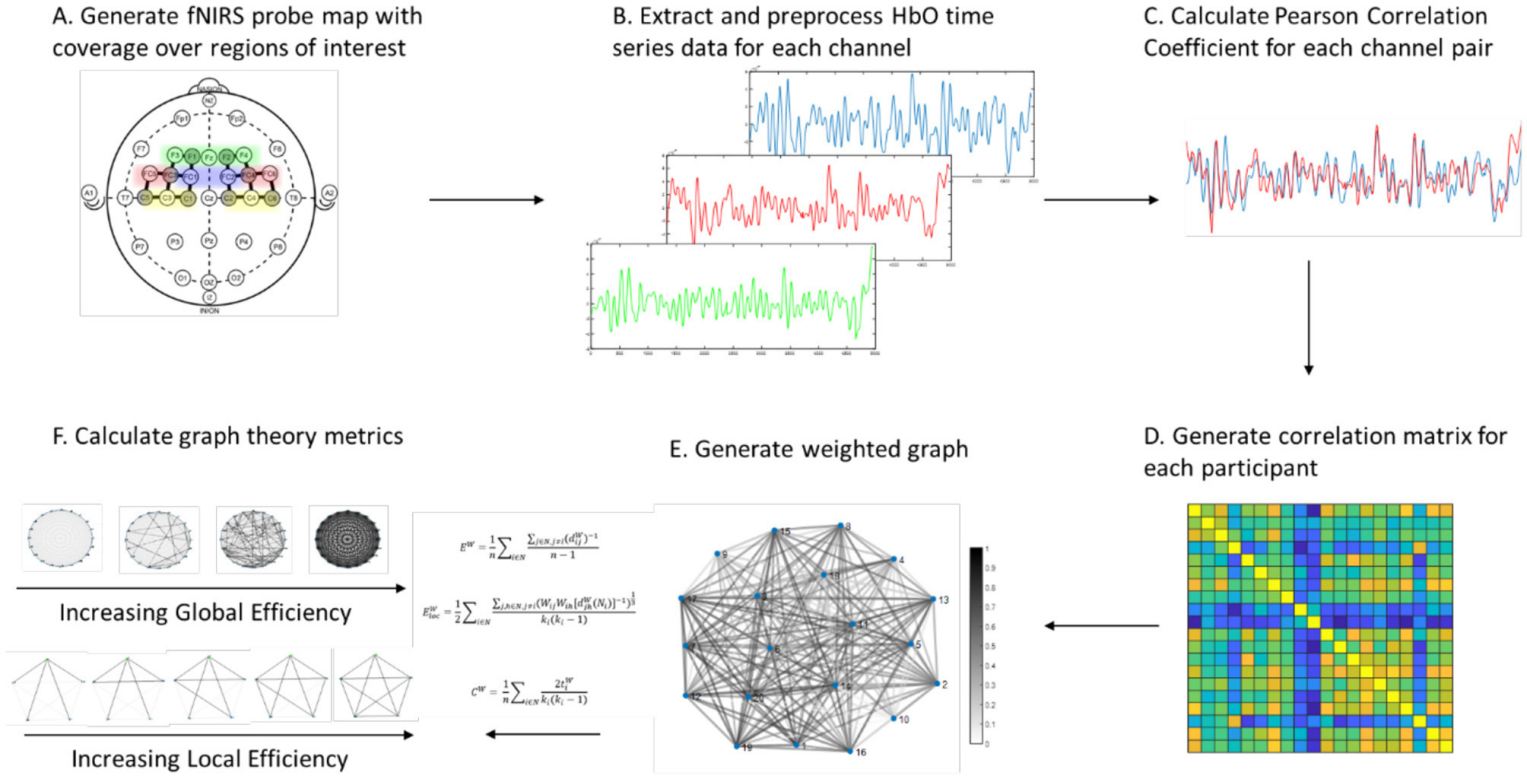
Pipeline for generation of graphs from fNIRS data and calculation of graph theory metrics.

Graph theoretical analysis (GTA) is a network-level analysis that provides information about the functional integration and segregation of different brain regions during a task (Rubinov and Sporns, 2010). In GTA, the brain is analyzed as a complex network consisting of nodes connected by edges. Each node represents an fNIRS channel, and each edge represents the correlation in HbO signals of the two nodes connected to that edge. For each participant, a weighted, undirected graph was generated in MATLAB using the un-thresholded Pearson Correlations for each channel pair. Unthresholded analyses were chosen to minimize threshold biases (Drakesmith et al., 2015). Global efficiency, local efficiency, clustering coefficient, and modularity were calculated for each graph using the MATLAB *Brain Connectivity Toolbox* (Rubinov and Sporns, 2010) (see Figure 2).

Prior studies have found increased clustering coefficient associated with motor learning and skill acquisition (Heitger et al., 2012) and distinct changes in modularity, global efficiency, and local efficiency associated with motor and cognitive tasks (Cohen and D'Esposito, 2016). Global efficiency reflects the functional integration of the brain, while local efficiency, clustering coefficient, and modularity reflect the functional segregation and community structure of brain networks (Rubinov and Sporns, 2010). Together, these metrics provide insight into the efficiency of communication within the brain and the neural strategies employed by a participant while performing a task.

Modularity was calculated using the Louvain community detection algorithm (Blondel et al., 2008). Global efficiency (Equation 1), local efficiency (Equation 2), and clustering coefficient (Equation 3) were calculated using the formulas below, where *N* represents the set of all of the nodes in the graph, *n* is the total number of nodes, *(i, j)* represents an edge between the nodes *i* and *j, w*_*i, j*_ represents the weight of the edge *(i, j), l*^*W*^ represents the sum of the weights of all edges, $k_{i}^{-}$ represents the degree of the node *i, t*_*i*_ represents the number of triangles around node *i*, and $d_{ij}^{W}$represents the shortest weighted path length between nodes *i* and *j* (Rubinov and Sporns, 2010).

Global Efficiency:


(1) $$\begin{array}{l} E^{W}=\frac{1}{n}\sum_{i\in N}\frac{\sum_{j\in N,\;j\neq i}{\left(d_{ij}^{W}\right)}^{-1}}{n-1} \end{array}$$

Local Efficiency:


(2) $$\begin{array}{l} E_{loc}^{W}=\frac{1}{2}\sum_{i\in N}\frac{\sum_{j,\;h\in N,\;j\neq i}{\left(W_{ij}W_{ih}{\left[d_{jh}^{W}(N_{i})\right]}^{-1}\right)}^{\frac{1}{3}}}{k_{i}(k_{i}-1)} \end{array}$$

Clustering Coefficient:


(3) $$\begin{array}{l} C^{W}=\;\frac{1}{n}\sum_{i\in N}\frac{{2t}_{i}^{W}}{k_{i}\left(k_{i}-1\right)} \end{array}$$

### 2.3 Statistical analysis

All statistical analysis was performed in the coding language R, version 4.1.1. All dependent measures were tested for normality with the Shapiro-Wilk normality test (Shapiro and Wilk, 1965), and three sets of statistical analyses were performed. First, we employed the Spearman Rank Correlation analysis to assess the relationships between neural activation and task performance measures for each interaction. Second, we evaluated the impact of training modality and sex on the time of completion for each interaction during the evaluation. For interactions with normal distributions, we performed a two-way between-subjects ANOVA for the effects of group (AR vs. video) and sex. For interactions with non-normal distributions, we performed an aligned ranks non-parametric two-way between-subjects ANOVA using the R package *ARTool* (Wobbrock et al., 2011).

Second, to examine changes in subjective workload and user experience, both between the two training modalities and over time, we analyzed the subjective survey responses for the main effects of group (AR vs. video), phase (training vs. evaluation), and sex for each interaction. A non-parametric test for factorial experiments with longitudinal data was performed, using the R package *nparLD* (Noguchi et al., 2012), because it has shown to be robust to non-continuous data. This test sets the denominator degrees of freedom as infinity for ANOVA-type statistics, so results from this analysis are reported with only the numerator degrees of freedom (Noguchi et al., 2012). *Post hoc* tests were conducted using the R package *nparcomp* (Konietschke et al., 2015).

Third, to compare differences in brain function during the evaluation task, we analyzed the activation, connectivity, and graph theory metrics for the effects of group (AR vs. video) and sex. Due to the variable nature of physiological data, values falling outside of the first and third quartile by more than one and a half times the interquartile range were considered outliers and removed from the analysis for the fNIRS data. For normally distributed measures, a two-way between-subjects ANOVA was performed. For non-normally distributed measures, an aligned ranks non-parametric two-way between subjects ANOVA was performed using the R package *ARTool* (Wobbrock et al., 2011). *Post hoc* tests were conducted using the “pairwise_t_test” function within the R package *rstatix* (Kassambara, 2019) for normal data and the R package *ARTool* (Elkin et al., 2021) for non-normal data.

Across all analyses, Bonferroni corrections were applied to account for multiple comparisons.

## 3 Results

All participants completed their assigned training and the evaluation task. Table 1 provides the mean (SD) of the performance and subjective responses (i.e., NASA-TLX, CLT, and UES scores) across both groups and sexes and for the training and evaluation phases. No significant correlations were found between neural activation of the different brain regions and task performance during poking, raycasting, or moving interactions (all *p's* > 0.123). However, scrolling performance was positively correlated with HbO levels at the RPMC (ρ = 0.373; *p* = 0.04).

**Table 1:** Mean (SD) of performance during the evaluation task and subjective responses to cognitive load theory (CLT), NASA Task Load Index (NASA-TLX), and user engagement survey (UES) across phases, groups, and type of interaction.

			**Training**		**Evaluation**	
			**AR**	**Video**	**AR**	**Video**
Performance	Time of completion (s)	Poking^G^			15.56 (3.56)	23.64 (7.09)
		Raycasting^G^			28.73 (10.18)	78.78 (66.89)
		Scrolling^G^			22.38 (11.59)	62.02 (32.13)
		Moving^G^			9.84 (4.93)	16.33 (8.94)
CLT	Germane	Poking^G^	4.88 (1.41)	6.42 (0.81)	5.17 (1.62)	6.6 (0.56)
		Raycasting^G^	4.77 (1.4)	6.33 (0.72)	5.35 (1.72)	6.48 (0.81)
		Scrolling^G^	4.92 (1.74)	6.4 (0.8)	5.02 (1.52)	6.54 (0.73)
		Moving^G^	5 (1.66)	6.19 (1.19)	5.12 (1.73)	6.79 (0.4)
	Intrinsic	Poking	1.78 (0.73)	1.94 (0.87)	1.69 (0.93)	1.84 (1.03)
		Raycasting^GxP^	3.44 (1.59)	2.12 (1.24)	2.56 (1.31)	3.31 (1.67)
		Scrolling^GxP^	3.72 (1.46)	2.31 (1)	2.28 (1.02)	3.41 (1.99)
		Moving	2.22 (1.38)	2.16 (1.23)	2.31 (1.61)	1.97 (1.22)
	Extraneous	Poking	1.35 (0.49)	1.21 (0.47)	1.44 (0.54)	1.23 (0.38)
		Raycasting^GxP^	2.75 (1.74)	1.6 (0.88)	1.85 (1.06)	2.52 (1.76)
		Scrolling^GxP^	2.6 (1.28)	1.65 (0.89)	1.96 (1.13)	2.35 (1.58)
		Moving	1.46 (0.56)	1.73 (0.9)	1.71 (0.95)	1.46 (0.53)
	Overall	Poking^G^	2.67 (0.56)	3.19 (0.34)	2.76 (0.68)	3.23 (0.41)
		Raycasting^GxP^	3.65 (1.37)	3.35 (0.7)	3.26 (0.91)	4.1 (0.78)
		Scrolling^GxP^	3.75 (1.09)	3.45 (0.5)	3.09 (0.67)	4.1 (0.96)
		Moving	2.89 (0.91)	3.36 (0.44)	3.05 (1)	3.41 (0.44)
NASA-TLX	Mental demand	Poking	2.75 (2.35)	3 (4)	2.31 (1.58)	3.18 (2.72)
		Raycasting^GxP^	7.44 (5.49)	2.94 (2.56)	3.56 (3.16)	6.24 (4.99)
		Scrolling^GxP^	7.44 (4.03)	3.29 (2.8)	3.25 (2.86)	8.53 (5.58)
		Moving	2.75 (2.84)	2.94 (2.66)	2.38 (1.86)	3.06 (2.97)
	Physical demand	Poking^GxP^	3.06 (2.29)	2.59 (4.81)	2.06 (1.48)	3.24 (3.21)
		Raycasting^GxP^	5.81 (4.39)	2.65 (3.37)	2.81 (2.59)	4.94 (4.66)
		Scrolling^GxP^	4.75 (4.09)	2.29 (2.54)	2.88 (3.61)	5.47 (5.16)
		Moving	2.25 (2.84)	2.06 (2.51)	1.75 (1.34)	2.53 (2.12)
	Temporal Demand	Poking	3 (2.45)	2.24 (2.28)	3.06 (4.67)	1.71 (1.05)
		Raycasting	4.56 (3.31)	2.71 (2.85)	3.56 (3.92)	2.71 (2.39)
		Scrolling	3.12 (2.7)	2.59 (2.09)	4 (4.97)	2.41 (2.29)
		Moving	2.56 (3.03)	3.47 (4.19)	2.94 (4.65)	2.18 (2.24)
	Performance	Poking	2.25 (1.77)	1.59 (2.18)	2.88 (4.49)	1.76 (1.82)
		Raycasting^GxP^	5.75 (3.8)	1.47 (0.87)	2.38 (1.78)	4.71 (4.41)
		Scrolling^GxP^	5.94 (4.2)	2 (2.03)	3.56 (3.52)	5.29 (4.36)
		Moving	3.69 (4.39)	2.29 (2.54)	2.25 (2.35)	2.06 (2.16)
	Effort	Poking	3.94 (2.89)	3.41 (4.14)	2.88 (2.55)	3.82 (3.3)
		Raycasting	10.12 (5.9)	3.71 (3.41)	5 (4.77)	10.59 (6.79)
		Scrolling^GxP^	10.19 (5.33)	4.12 (3.82)	6.25 (5.37)	12.18 (5.65)
		Moving	3.69 (3.7)	3.53 (3.47)	2.75 (2.46)	3.76 (3.83)
	Frustration	Poking	2.81 (3.69)	1.76 (1.39)	2.25 (3.3)	1.94 (1.52)
		Raycasting^GxP^	7.25 (6.44)	1.94 (1.68)	4.69 (6.13)	4.94 (4.53)
		Scrolling^GxP^	5.5 (5.05)	2.12 (2.06)	3.56 (4.1)	6.59 (5.03)
		Moving	2.44 (3.01)	1.71 (1.57)	1.94 (2.26)	2.06 (1.34)
	Total workload	Poking	17.81 (11.33)	14.59 (17.41)	15.44 (10.73)	15.65 (10.95)
		Raycasting^GxP^	40.94 (24.79)	15.41 (12.27)	22 (16.38)	34.12 (21.08)
		Scrolling^GxP^	36.94 (18.62)	16.41 (11.79)	23.5 (17.88)	40.47 (22)
		Moving	17.38 (15.62)	16 (14.65)	14 (9.22)	15.65 (10.98)
UES	Focused attention^P^		2.58 (0.61)	2.29 (0.63)	3 (1.02)	2.73 (0.86)
	Perceived usability^GxP^		3.67 (0.92)	4.73 (0.36)	3.94 (0.98)	3.98 (0.61)
	Aesthetic appeal		3.25 (1.06)	2.73 (0.98)	3.54 (1.21)	3.43 (1.2)
	Reward		3.83 (0.89)	3.78 (0.76)	3.96 (0.96)	3.67 (1.09)
	Overall engagement		3.33 (0.59)	3.38 (0.49)	3.61 (0.82)	3.45 (0.71)

^G,P,GxP^ denote significant group and phase main effects, and group x phase interaction effects, respectively.

### 3.1 Performance

**Figure 3:**
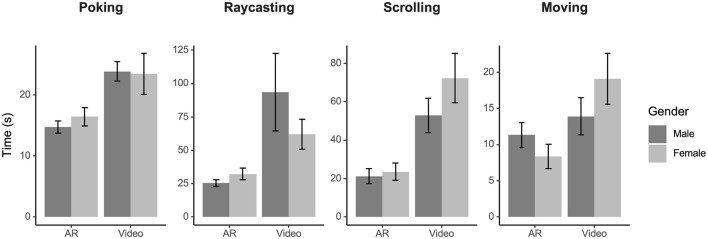
Performance during the evaluation task by sex, group, and interaction plotted as time of completion in seconds. The AR group performed better than the video group for all four interactions (all *p* < 0.05). There were no main effects of sex (all *p* > 0.20) or interaction effects of group and sex (all *p* > 0.07). Error bars represent standard error.

The time of completion (Figure 3) for the AR group was significantly lower than the video group for poking [Group Main Effect: *F*_(1, 29)_ = 22.66, *p* < 0.001, $\eta_{p}^{2}$ = 0.44], raycasting [Group Main Effect: *F*_(1, 29)_ = 27.07, *p* < 0.001, $\eta_{p}^{2}$ = 0.48], scrolling [Group Main Effect: *F*_(1, 29)_ = 22.81, *p* < 0.001, $\eta_{p}^{2}$ = 0.44], and moving [Group Main Effect: *F*_(1, 29)_ = 7.14, *p* = 0.01, $\eta_{p}^{2}$ = 0.20]. No sex effects (all *p* > 0.20) or interaction effect on group and sex (all *p* > 0.07) were identified.

### 3.2 Cognitive load theory

For the poking interaction, irrespective of phase or sex, participants in the video group reported greater germane load [Group Main Effect: *F*_(1)_ = 17.22, *p* < 0.001] and greater overall cognitive load [Group Main Effect: *F*_(1)_ = 17.47, *p* < 0.001] than the AR group (see Table 1).

For the raycasting interaction, irrespective of phase or sex, participants in the video group reported greater germane load [Group Main Effect: *F*_(1)_ = 11.24, *p* < 0.001] than the AR group. Additionally, there was an interaction effect on intrinsic load [Group x Phase Interaction: *F*_(1)_ = 12.92, *p* < 0.001], extraneous load [Group x Phase Interaction: *F*_(1)_ = 10.40, *p* = 0.001], and overall load [Group x Phase Interaction: *F*_(1)_ = 8.91, *p* = 0.003]. During training, the AR group reported higher intrinsic and extraneous load than the video group. During evaluation, the video group reported a greater overall load than the AR group. The video group reported higher intrinsic and overall load during evaluation compared to training (see Table 1).

For the scrolling interaction, irrespective of phase or sex, participants in the video group reported a greater germane load [Group Main Effect: *F*_(1)_ = 12.75, *p* < 0.001] than the AR group. Additionally, there was an interaction effect on intrinsic load [Group x Phase Interaction: *F*_(1)_ = 24.41, *p* < 0.001], extraneous load [Group x Phase Interaction: *F*_(1)_ = 13.21, *p* < 0.001], and overall load [Group x Phase Interaction: *F*_(1)_ = 22.0, *p* < 0.001]. During training, the AR group reported greater intrinsic and extraneous load than the video group. During evaluation, the video group reported a greater overall load than the AR group. The AR group reported greater intrinsic and extraneous load during training than during evaluation (see Table 1).

For the moving interaction, irrespective of phase or sex, participants in the video group reported greater germane load [Group Main Effect: *F*_(1)_ = 17.39, *p* < 0.001] than the AR group (see Table 1).

### 3.3 NASA task load index

For the poking interaction, there was a group x phase interaction effect on physical demand (Group x Phase Interaction: *F*_(1)_ = 9.87, *p* = 0.002), wherein the video group reported greater levels during evaluation than during training (see Table 1).

For the raycasting interaction, there was a group x phase interaction effect on mental demand [Group x Phase Interaction: *F*_(1)_ = 29.21, *p* < 0.001], physical demand [Group x Phase Interaction: *F*(1) = 45.07, *p* < 0.001], effort [Group x Phase Interaction: *F*_(1)_ = 41.37, *p* < 0.001], performance [Group x Phase Interaction: *F*_(1)_ = 38.96, *p* < 0.001], frustration [Group x Phase Interaction: *F*_(1)_ = 18.92, *p* < 0.001], and total workload [Group x Phase Interaction: *F*_(1)_ = 45.15, *p* < 0.001]. During training, the AR group reported higher mental demand, physical demand, effort, performance, frustration, and overall workload than the video group. During evaluation, the video group reported greater effort than the AR group. The AR group reported greater mental demand, physical demand, effort, performance, and total workload during training than during evaluation. The video group reported greater mental demand, physical demand, effort, performance, frustration, and total workload during evaluation than during training (see Table 1).

For the scrolling interaction, there was a group x phase interaction effect on mental demand [Group x Phase Interaction: *F*_(1)_ = 44.17, *p* < 0.001], physical demand [Group x Phase Interaction: *F*_(1)_ = 39.90, *p* < 0.001], effort [Group x Phase Interaction: *F*_(1)_ = 45.13, *p* < 0.001], performance [Group x Phase Interaction: *F*_(1)_ = 13.74, *p* < 0.001], frustration [Group x Phase Interaction: *F*_(1)_ = 22.50, *p* < 0.001], and total workload [Group x Phase Interaction: *F*_(1)_ = 41.05, *p* < 0.001]. During training, the AR group reported greater mental demand, physical demand, effort, performance, frustration, and total workload than the video group. During evaluation, the video group reported greater mental demand and effort than the AR group. The AR group reported greater mental demand, physical demand, effort, performance, and total workload during training than during evaluation. The video group reported greater mental demand, physical demand, effort, frustration, and total workload during evaluation than during training (see Table 1).

No significant main effects or interaction effects were found for the moving interaction (see Table 1).

### 3.4 User engagement survey

Irrespective of group or sex, participants reported higher focused attention [Phase Main Effect: *F*_(1)_ = 8.09, *p* = 0.005], during the evaluation compared to the training, but they rated perceived usability [Phase Main Effect: *F*_(1)_ = 11.29, *p* < 0.001] higher in the training than in the evaluation. There was a group x phase interaction effect for perceived usability [Group x Phase Interaction: *F*_(1)_ = 20.16, *p* < 0.001]. During training, the video group reported higher perceived usability than the AR group, and the video group reported higher perceived usability during training than during evaluation (see Table 1).

### 3.5 Brain activation

**Figure 4:**
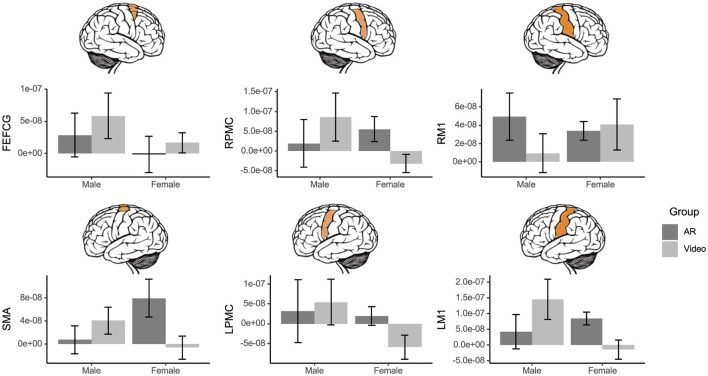
Mean brain activation by group, sex, and brain region, measured as the change in HbO concentration from baseline, captured by fNIRS during the poking evaluation. A group x sex interaction was identified for the SMA (*p* = 0.026) and the LM1 (*p* = 0.043). Females in the AR group displayed higher activation of SMA and LM1 than females in the video group. Error bars represent standard error.

For the poking interaction, there was a two-way interaction effect for mean activation of the SMA [Group x Sex Interaction: *F*_(1, 26)_ = 5.59, *p* = 0.026, $\eta_{p}^{2}$ = 0.18] and LM1 [Group x Sex Interaction: *F*_(1, 23)_ = 4.58, *p* = 0.043, $\eta_{p}^{2}$ = 0.17]. Females in the AR group displayed higher activation of the SMA and LM1 than females in the video group (Figure 4).

During raycasting, no significant group effects, sex effects, or interaction effects of group and sex (all *p* > 0.17) were identified in the FEF/CG (Mean: 1.36e-08; SD: 3.53e-08), SMA (Mean: 9.86e-09; SD: 4.21e-08), LPMC (Mean:−2.31e-09; SD: 9.75e-08), RPMC (Mean: 3.08e-09; SD: 5.96e-08), LM1 (Mean: 1.53e-09; SD: 6.99e-08), or RM1 (Mean: 1.95e-08; SD: 4.51e-08).

**Figure 5:**
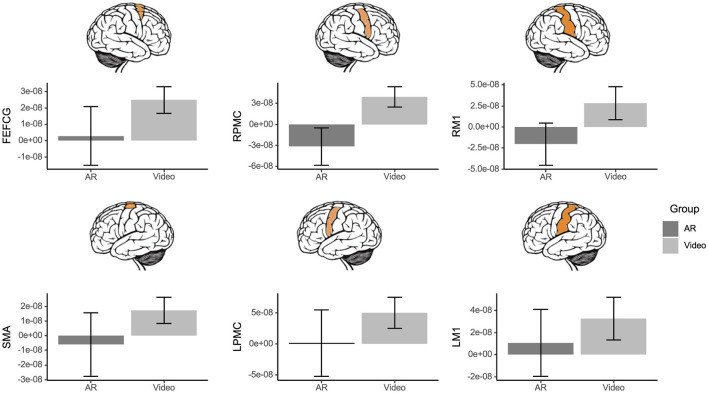
Mean brain activation by group and brain region, measured as the change in HbO concentration from baseline, captured by fNIRS during the scrolling evaluation. The AR group displayed lower RPMC activation than the video group (*p* = 0.038). Error bars represent standard error.

During the scrolling interaction, irrespective of sex, the AR group displayed lower RPMC activation than the video group [Group Main Effect: *F*_(1, 25)_ = 4.80, *p* = 0.038, $\eta_{p}^{2}$ = 0.16; Figure 5], while no other ROI was influenced by group and sex effects (all *p* > 0.05).

During moving, no significant group effects, sex effects, or interaction effects of group and sex (all *p* > 0.12) were identified in the FEF/CG (Mean: 1.45e-09; SD: 1.96e-07), SMA (Mean:−2.2e-09; SD: 1.62e-07), LPMC (Mean: 5.76e-08; SD: 2.26e-07), RPMC (Mean:−3.07e-09; SD: 2.31e-07), LM1 (Mean: 8.48e-08; SD: 1.78e-07), or RM1 (Mean: 3.27e-08; SD: 2.11e-07).

### 3.6 Functional connectivity

**Figure 6:**
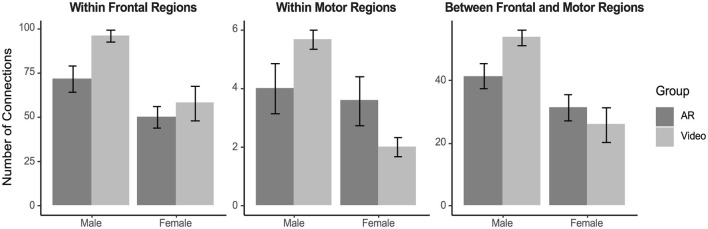
Number of non-spurious channel-wise connections identified in the brain during the evaluation task, plotted by group and sex. Males displayed more connections than females within the frontal regions (*p* < 0.001). Males displayed more connections than females within the motor regions (*p* = 0.017) and there was a group x sex interaction within the motor regions (*p* = 0.013). Within the video group males displayed more connections within the motor regions than females, while there was no sex difference in the AR group. Males displayed more connections between the frontal and motor regions than females (*p* < 0.001). Error bars represent standard error.

Within the frontal regions, irrespective of group, males displayed more connections than females [Sex Main Effect: *F*_(1, 24)_ = 15.38, *p* < 0.001, $\eta_{p}^{2}$ = 0.39]. Within the motor regions, there was a two-way interaction effect [Group x Sex Interaction: *F*_(1, 24)_ = 7.24, *p* = 0.013, $\eta_{p}^{2}$ = 0.23; Figure 6] in which males in the video group displayed more connections than females in the video group, while no sex differences were observed in the AR group. Between the frontal and motor regions, males displayed more connections than females [Sex Main Effect: *F*_(1, 25)_ = 18.31, *p* < 0.001, $\eta_{p}^{2}$ = 0.42].

### 3.7 Graph theory metrics

**Figure 7:**
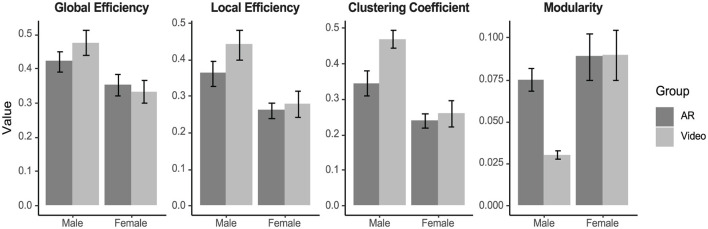
Graph theory metrics (global efficiency, local efficiency, clustering coefficient, and modularity) generated from weighted graphs of brain connectivity (i.e., channel-wise Pearson Correlation) during the evaluation task, plotted by group and sex. Males exhibited higher global efficiency (*p* = 0.004), local efficiency (*p* < 0.001), and clustering coefficient (*p* < 0.001) than females, and males exhibited lower modularity than females (*p* = 0.004). Error bars represent standard error.

Males exhibited a higher global efficiency [Sex Main Effect: *F*_(1, 26)_ = 10.27, *p* = 0.004, $\eta_{p}^{2}$ = 0.28], local efficiency [Sex Main Effect: *F*_(1, 25)_ = 14.03, *p* < 0.001, $\eta_{p}^{2}$ = 0.36], and clustering coefficient [Sex Main Effect: *F*_(1, 24)_ = 22.30, *p* < 0.001, $\eta_{p}^{2}$ = 0.48] than females, and males exhibited lower modularity than females [Sex Main Effect: *F*_(1, 24)_ = 10.48, *p* = 0.004, $\eta_{p}^{2}$ = 0.30]. Figure 7 illustrates the sex effects.

## 4 Discussion

The goal of this study was to compare the effectiveness of AR- and video-based training for motor learning and to use neurophysiological and subjective data to understand and explain potential differences in performance between the two modalities. Our main findings are summarized as follows:

1. AR-based psychomotor training places greater perceived demands on the user during training but leads to improved performance during evaluation.
2. Neural data suggests that learners in the AR group utilize a more efficient brain network during the evaluation task and that the impacts of AR training are interaction-specific.
3. Sex differences in brain activation and connectivity patterns suggest distinct neural strategies associated with psychomotor learning, which could contribute to differences in user experience.

### 4.1 AR-based psychomotor training places greater perceived demands on the user during training but leads to improved performance during evaluation

During the evaluation, participants in the AR group exhibited faster completion times than those in the video group across all four interactions. Furthermore, participants in the video group showed more variability in performance (i.e., a larger standard deviation in evaluation completion times) than those in the AR group. These findings indicate that hands-on AR-based psychomotor learning is more effective and consistent than video-based observational learning for teaching these basic AR interactions, leading to two major implications. First, when introducing AR to a new group of users, they should receive hands-on training in AR rather than video-based instruction for basic AR interactions to expedite skill acquisition and diminish the learning curve associated with early AR use. This will enable users to become comfortable with the AR interface more quickly, allowing them to use AR more effectively during contextual training and in practical applications. In this study, we found that just six trials within the AR headset were sufficient to enhance skill acquisition in the AR group compared to the video group. This raises the question of how much practice is necessary for effective skill acquisition. While the specific number of trials is likely dependent on the individual (Vyas et al., 2024), it is possible that the AR training could be even further reduced while still maintaining the benefits over the video modality. Second, these findings suggest that AR-based motor learning could be more effective than video-based observational learning in a wide variety of training applications. Future studies should explore whether these findings extend to learning industry-relevant skills in AR (e.g., triage) (Nelson et al., 2022) rather than basic AR interaction skills (such as those evaluated here). It is also important to note that this study focused specifically on learning basic AR interaction skills and not skills that would transfer to an analogous task in the real world. Future studies should explore whether practicing a real-world skill virtually in an AR simulation leads to improved performance of the actual skill in the real world, which has important technology acceptance considerations for fielded AR applications (Mehta et al., 2022).

A relatively consistent pattern associated with perceived workload (as measured by NASA-TLX) was observed during the raycasting and scrolling interactions. During training, the AR group reported a higher workload, but during evaluation, the AR group perceived a significant decrease in workload. Conversely, the video group experienced a lower workload during training but displayed a significant increase moving into evaluation. These findings suggest that the increased demands during training perceived by the AR group may have potentially increased their arousal and engagement, leading to improved learning and better performance during evaluation. Indeed, it has been found that mild levels of stress and arousal can help to improve performance (Teigen, 1994), which is consistent with our results. Interestingly, this pattern was only observed in raycasting and scrolling but not in poking or moving. Different interactions likely place different demands on the user, and it has been found previously that the design of the AR interface can significantly induce mental and physical demands (Kia et al., 2021). Raycasting has indeed been found to be a more complex visuomotor interaction (Argelaguet and Andujar, 2013), displaying a non-linear relationship between the target's angular size and the task's difficulty (Kopper et al., 2010). This has important implications for the design of future AR interfaces. A thorough understanding of the impacts of different AR tasks and interactions on user experience and perceptions must guide the design of such interfaces. Compared to the AR group, the video group perceived very low demands during training, which is expected, but failed to effectively acquire the skills needed for efficient performance, leading to longer completion times and greater workload during the evaluation.

In addition to perceived workload, which is a major determinant of learning and skill acquisition (Akizuki and Ohashi, 2015), it is also important to examine through the lens of Cognitive Load Theory (Klepsch et al., 2017) whether different training or instructional modalities (AR vs video) facilitate learning. A recent systematic review of research on AR and CLT has emphasized the need to assess the different loads (germane, intrinsic, and extraneous) placed on users when using AR-based instructional modalities (Buchner et al., 2021), which the present study captured. Optimizing intrinsic load (i.e., the load associated with the complexity of a task, which is influenced by both the amount of information that must be maintained in working memory during the task and by the prior knowledge of the user) is a goal in developing effective instructional materials (Klepsch et al., 2017). In the present study, the AR group reported a higher intrinsic load than the video group for raycasting and scrolling during training. This finding, which suggests that watching a video is intrinsically easier than completing a complex and unfamiliar motor task, is not surprising. Interestingly, intrinsic load decreased in the AR group moving into the evaluation task for the scrolling task. This indicates that AR training effectively imparted the skills/knowledge needed to perform the task, allowing the participants to rely on recently acquired knowledge/experience to reduce the intrinsic load of the task during evaluation.

It is important to develop training that minimizes the extraneous load associated with design elements of a learning task that distracts the user from learning (Klepsch et al., 2017). During training, the AR group reported a higher extraneous load than the video group for raycasting and scrolling. One training element likely contributing to this difference was the text-based and verbal instructions. Participants in the video group received written and verbal instructions followed by a video demonstration. In contrast, participants in the AR group only received written and verbal instructions and were then required to perform each interaction based solely on these instructions. This approach forced the AR group to determine each interaction based on the written guidelines and a trial-and-error method, rather than being able to clearly visualize how each interaction was performed. Despite this challenge, the extraneous load in the AR group decreased during the evaluation for the scrolling task. This suggests that while the novelty of the interface and the unfamiliarity with the interactions initially contributed to the extraneous load, it diminished as participants became more accustomed to the interactions. Future training could integrate a short video demonstration with hands-on practice to minimize extraneous load.

It was interesting and surprising to note that germane load was rated higher in the video group than in the AR group across both phases and all four interactions. According to cognitive load theory, germane load is the type of cognitive load that enhances learning and aids in the development of mental models or schemas to store and organize new information (Klepsch et al., 2017). One might expect that a greater germane load during training would enhance performance during evaluation; however, in this study, the video group underperformed despite the increased germane load. Previous studies have found that dynamic visualizations (i.e., videos) can be highly effective at inducing increased germane load over static visualizations (Ayres and Gog, 2009), which helps to explain the high levels of germane load associated with video training in the current study. Based on the findings of this study, we propose that the increased germane load from video training helped participants achieve a better conceptual understanding and develop a mental model for the interactions (i.e., the position of the hand, what each finger should do, etc.); however, it fell short in providing the actual hands-on practice and motor training that the AR modality offered. Future studies should investigate the combination of video and AR training modalities to optimize germane load and enhance user performance.

The video group reported higher perceived usability on the user engagement scale compared to the AR group during training. This finding aligns with the lower frustration associated with video-based training and the reduced intrinsic load of watching a video. Interestingly, during evaluation, despite performance differences between the two groups, both reported similar levels of perceived usability. Additionally, focused attention was noted to be higher during evaluation, regardless of the group. This suggests that while skill acquisition and performance during evaluation are largely influenced by training modality, user engagement in AR during evaluation remains mostly unaffected by the training modality used.

### 4.2 Neural data suggests that participants in the AR group utilize a more efficient brain network during the evaluation task and that the impacts of AR training are interaction-specific

Previous studies have linked decreased activation of the premotor cortex with motor learning and the development of automaticity (Toni et al., 1998; Debaere et al., 2004; Wu et al., 2004), and the difference in RMPC activity suggests that participants in the AR group learned the interaction better than participants in the video group. The premotor cortex is involved in motor planning (Brovelli et al., 2005), so it is possible that participants in the AR group required less planning and completed the interaction more automatically. Participants in the video group had a conceptual understanding of what the interaction required (indicated by the high germane load) but required greater engagement of the premotor cortex to implement this action.

It is unclear why this change was inconsistent across interactions, given that performance was better in AR across all four interactions. This may potentially be related to the difficulty of the interactions (Wu et al., 2004). Indeed, differences in performance across groups were greater for raycasting and scrolling than for poking and moving. Additionally, raycasting and scrolling revealed differences in cognitive load and subjective workload between groups during evaluation that were not observed during poking and moving. This is further supported by the positive correlation between scrolling performance and activation of the premotor cortex. Collectively, these findings suggest that the benefits of AR training are interaction-specific, with greater advantages on tasks that are more complex or challenging. However, it is important to note that limitations in the analysis technique may have also contributed to the differences in activation patterns between interactions. Activation analysis from hemodynamic data requires highly dynamic time-series data to be condensed into a single data point. Based on the design of this study, mean activation was chosen as the best option for this analysis, but this decision comes with limitations and could have muted some of the effects due to averaging.

Our study findings contradict a previous study that reported an increased clustering coefficient with improved motor performance (Heitger et al., 2012). However, the mentioned study used fMRI and, therefore, a different set of nodes for the graph theoretical analysis. This could lead to differences in the interpretation of the results, as it is recommended that graphs should only be compared when generated with the same parcellation scheme (Rubinov and Sporns, 2010). Our findings may indicate a form of functional reorganization or “pruning” (Kelly and Garavan, 2004) in the AR group that led to the recruitment of a more efficient brain network to complete the task. Prior literature suggests that early in the process of learning a new task, additional brain regions are activated, usually within the PFC, to support the novel demands of a new task (Petersen et al., 1998). As a person practices and becomes more familiar with the task, these additional regions are pruned away, leaving activation of only the more essential regions (Petersen et al., 1998; Kelly and Garavan, 2004). As non-essential regions fall away, connections would decrease, leaving a more efficient network with a smaller footprint, such as that observed with the AR group in the present study. While scaffolding is generally associated with the activation of regions of the PFC associated with attention and control (Kelly and Garavan, 2004), it is possible that it also occurs in other frontal regions contained within our fNIRS probe maps, such as the premotor cortex and frontal eye fields.

### 4.3 Sex differences in brain activation and connectivity suggest different neural strategies across sex, which could contribute to differences in user experience

Sex differences in the functional connectivity and graph theory metrics further support the concept that males and females employ different neural strategies during motor learning. While these differences may be an artifact of the brain imaging methodology (i.e., they reflect differences in vasculature between males and females and not differences in neural activity), sex differences in neural activity have been previously observed in both motor and cognitive tasks across a range of neuroimaging techniques that rely on different mechanisms (e.g., fNIRS, EEG, fMRI) (Tyagi and Mehta, 2024; Davidson et al., 1976; Lissek et al., 2007; Kober and Neuper, 2011). Interestingly, within the motor regions, a sex difference in functional connectivity was only observed in the video group and not in the AR group. This might indicate that, to some extent, AR training reduces differences in the neural strategies employed by males and females compared to video training. Further research is needed to explore the implications of this finding.

Understanding these differences is essential in developing equitable training tools that support training for both men and women (Keri, 2002). While the interaction-based training in this study was relatively easy, future applied training within AR directed toward real contexts (e.g., medical, industrial, educational) could place much greater demands on users, exacerbating sex-based differences in frustration, which could lead to differences in performance outcomes and differences in user experience. Further studies into sex-based differences in motor learning and training within AR are essential to identify and address such differences.

### 4.4 Study limitations

In this study, participants learned virtual skills (i.e., selection-based AR interactions) and were tested by completing those interactions. The main reason was to identify neurophysiological and perceptual differences specific to training modalities, aiming to better understand fundamental skill acquisition in key AR interactions, free from any contextual confounds noted in earlier work (Dwivedi et al., 2022). This study assessed participants trained using video and AR-based modalities through AR interactions. There is a need for more innovative experimental designs that tackle the biases associated with modality-specific assessments (e.g., evaluating video training with video assessments). More importantly, future studies should explore transferring the basic AR interaction skills learned in this study to more contextual interactions within AR (e.g., industry-specific training or field applications).

fNIRS is limited to measuring activation in the cortex, so we cannot observe how changes in the activation of subcortical structures contribute to motor learning during this study. Correlation analyses between task performance and brain region activation did not yield conclusive results, revealing only one significant positive relationship between scrolling task performance and activation of the premotor cortex. These findings highlight the necessity for further analyses with larger sample sizes and stress the importance of exploring network analyses beyond activation levels. Since the time of completion for the study was used as a performance metric, the duration of each trial varied. Ideally, each trial should have the same duration for fNIRS activation analysis, so this should be considered when interpreting the results. The moving interaction, in particular, was very brief, which could have affected the fNIRS measurements. Participants were given a rest period between interactions, but not after every trial, which could impact the fNIRS measurements of later trials, as HbO levels did not have enough time to return to baseline before the trials began. Prior studies have examined changes in the prefrontal cortex associated with motor learning (Wu et al., 2004; Alves Heinze et al., 2019), and this could have provided valuable insight into the cognitive demands associated with performing the interactions in AR. Unfortunately, the shape of the AR headset interfered with the placement of the prefrontal fNIRS probes. Future studies should identify ways to integrate fNIRS probes with the AR headset design to acquire prefrontal cortex measurements.

Connectivity and graph theoretical analyses require determining thresholds to reduce the risk of spurious effects. Thresholding for GTAs has been a contentious topic (Drakesmith et al., 2015). In network analysis, thresholding can induce biases due to arbitrary values and the effort to connect thresholds to minimize false positive outcomes. However, identifying the appropriate threshold a priori is challenging, especially in exploratory topics like the one presented here. Furthermore, thresholding increases the likelihood of observing false negatives in the network connections. There are methods (reviewed by Hosseini et al., 2012) to mitigate these biases (i.e., comparing network metrics across a range of thresholds). However, they may not support the current study's analyses. Future work is warranted to explore our analyses in light of the range of thresholds applied for GTA.

Lastly, future studies with larger and more diverse participant demographics should be conducted to expand upon the findings of this study and ensure generalizability across a broad range of user groups. For example, age can significantly impact motor performance through mechanisms impacting neural and peripheral pathways of neuromuscular functioning (Rhee and Mehta, 2018; Mehta and Rhee, 2021; Tyagi and Mehta, 2024). The participant age range in the study was diverse but not adequately powered to support formal analysis by age. Future work is needed to examine the impact of age on the study variables.

## 5 Conclusion

We found that participants who completed motor training within augmented reality performed better than those who completed video training. These findings should be considered when introducing AR into new industries and teaching new users how to interact with the interface. While prior studies have examined subjective workload and user experience when using augmented reality interfaces, we incorporated a novel neuroergonomics approach to identify the underlying neural mechanisms driving changes in psychomotor learning. Additionally, we identified significant sex differences in neural activity during evaluation, suggesting that men and women use different neural strategies when learning AR interactions. There is an urgent need to understand these differences when designing future AR training tools.

In this study, participants learned basic AR skills. Future studies should explore the use of AR for teaching more complicated and occupationally relevant skills (e.g., teaching triage to emergency responders). Additionally, future work should explore how effectively skills learned virtually in AR transfer to the real world. AR offers great potential to expedite and improve training in a vast range of fields, which can help reduce costs, improve safety, and increase the on-the-job effectiveness of those who are trained. While it is clear that the limitations of such training must be considered and understood, there is a huge potential for this technology to improve training.

## Data Availability

The raw data supporting the conclusions of this article will be made available by the authors, without undue reservation.
